# Supplementary material for: Unmet need of essential treatments for critical illness in Malawi
Source: PLoS One. 2021 Sep 10;16(9):e0256361. doi: 10.1371/journal.pone.0256361 (PMC8432792; doi:10.1371/journal.pone.0256361)
Supplement: S1 Table — (DOCX) [file pone.0256361.s001.docx]

Supplementary Table 1 Patient characteristics by hospital

|  | Chiradzulu District Hospital | Queen Elizabeth Central Hospital | Chi Square P Values |
| --- | --- | --- | --- |
|  | N=243 | N=892 |  |
| Female n (%) | 167 (68.7) | 506 (56.7) | 0.001 |
| Age, mean years(range) | 39 (18-91) | 39 (18-98) |  |
| HIV positive^*^, n (%) | 74/184 (40.2) | 293/662 (44.3) | 0.328 |
| Specialty, n (%)  Medicine  Surgery  Obstetrics and Gynaecology  Ophthalmology  Other | 109 (44.9)  57 (23.5)  76 (31.3)  0 (0)  1 (0.4) | 364 (40.8)  262 (29.4)  216 (24.2)  20 (2.2)  30 (3.4) | 0.004 |
| Hypotension n (%)  Hypoxia n (%)  Low conscious level n (%) | 23 (9.5)  13 (5.4)  5 (2.1) | 80 (9.0)  32 (3.6)  12 (1.4) | 0.831  0.212  0.418 |
| Unmet Need* n/N  Hypotension  Hypoxia  Low consciousness level | 23/23(100)  13/13(100)  2/5(40) | 74/80(92.5)  27/32(84.4)  7/12(58.3) | 0.908  0.098  0.961 |
| In hospital mortality n/N (%) | 9/242 (3.7) | 75/875 (8.6) | 0.011 |

^* unmet need refers to the number and proportion of patients with a deranged vital sign who were not receiving the needed, corresponding, essential treatment^
